# Complete and durable response of pulmonary large‐cell neuroendocrine carcinoma to pembrolizumab

**DOI:** 10.1002/cnr2.1589

**Published:** 2021-11-24

**Authors:** Naoki Kadota, Nobuo Hatakeyama, Hiroyuki Hino, Michihiro Kunishige, Yoshihiro Kondo, Yoshio Okano, Hisanori Machida, Keishi Naruse, Tsutomu Shinohara, Shoji Sakiyama, Fumitaka Ogushi, Eiji Takeuchi

**Affiliations:** ^1^ Department of Respiratory Medicine National Hospital Organization Kochi Hospital Kochi Japan; ^2^ Department of Thoracic Surgery National Hospital Organization Kochi Hospital Kochi Japan; ^3^ Department of Pathology National Hospital Organization Kochi Hospital Kochi Japan; ^4^ Department of Community Medicine for Respirology, Graduate School of Biomedical Sciences Tokushima University Tokushima Japan; ^5^ Department of Clinical Investigation National Hospital Organization Kochi Hospital Kochi Japan

**Keywords:** complete response, immune checkpoint inhibitor, pembrolizumab, plasma cell, pulmonary large cell neuroendocrine carcinoma

## Abstract

**Background:**

Pulmonary large cell neuroendocrine carcinoma (LCNEC) is a rare and aggressive tumor with a poor prognosis and standard therapy has not yet been established.

**Case:**

A 65‐year‐old male with a cough for 2 months presented to our hospital. He was clinically diagnosed with non small cell lung cancer cT3N1M0 stage IIIA and underwent right pneumonectomy. The final diagnosis was pulmonary LCNEC pT3N1M0 stage IIIA. Multiple subcutaneous masses were detected 4 months after surgery, and biopsy revealed postoperative recurrence and metastasis. Chemotherapy with carboplatin plus etoposide was initiated. Subcutaneous masses increased and multiple new brain metastases developed after two cycles. Additional tests revealed that epidermal growth factor receptor and anaplastic lymphoma kinase were negative, and the programmed death ligand 1 (PD‐L1) expression rate in tumor cells was 40% (22C3 clones). The primary cells infiltrating the tumor were CD3‐positive T cells and CD138‐positive plasma cells. Second‐line treatment with pembrolizumab was started. The shrinkage of subcutaneous masses was observed after one cycle, and the tumor had completely disappeared after six cycles. Treatment was continued for approximately 2 years. This response has been maintained for 4 years and is still ongoing.

**Conclusion:**

Pembrolizumab may be used as a treatment option for pulmonary LCNEC.

## 1 INTRODUCTION

Pulmonary large cell neuroendocrine carcinoma (LCNEC) is a rare and aggressive tumor that is diagnosed based on the high‐grade features of more than 10 mitotic figures in 2 mm^2^ and the presence of neuroendocrine markers, with characteristics of both small cell lung carcinoma (SCLC) and non small cell lung carcinoma (NSCLC).^1^ The prognosis of pulmonary LCNEC is poor and standard therapy has not yet been established.

Pembrolizumab is a humanized monoclonal antibody against programmed death 1 (PD‐1) with antitumor activity for advanced NSCLC.^2^, ^3^ However, limited information is currently available on the effectiveness of pembrolizumab for pulmonary LCNEC.^4^

We herein report a rare case of the complete response (CR) of pulmonary LCNEC to pembrolizumab.

## 2 CASE

**FIGURE 1:**
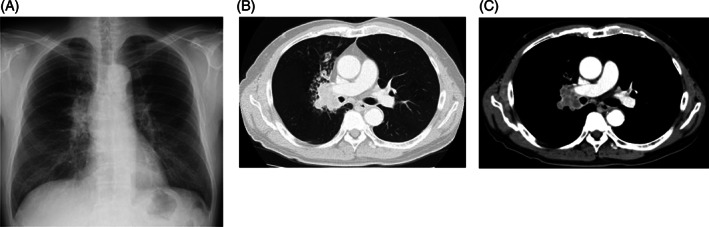
(A) Chest X‐ray and (B, C) contrast‐enhanced CT at the first admission. Chest X‐ray and contrast‐enhanced CT showed a tumor in the right upper lobe

**FIGURE 2:**
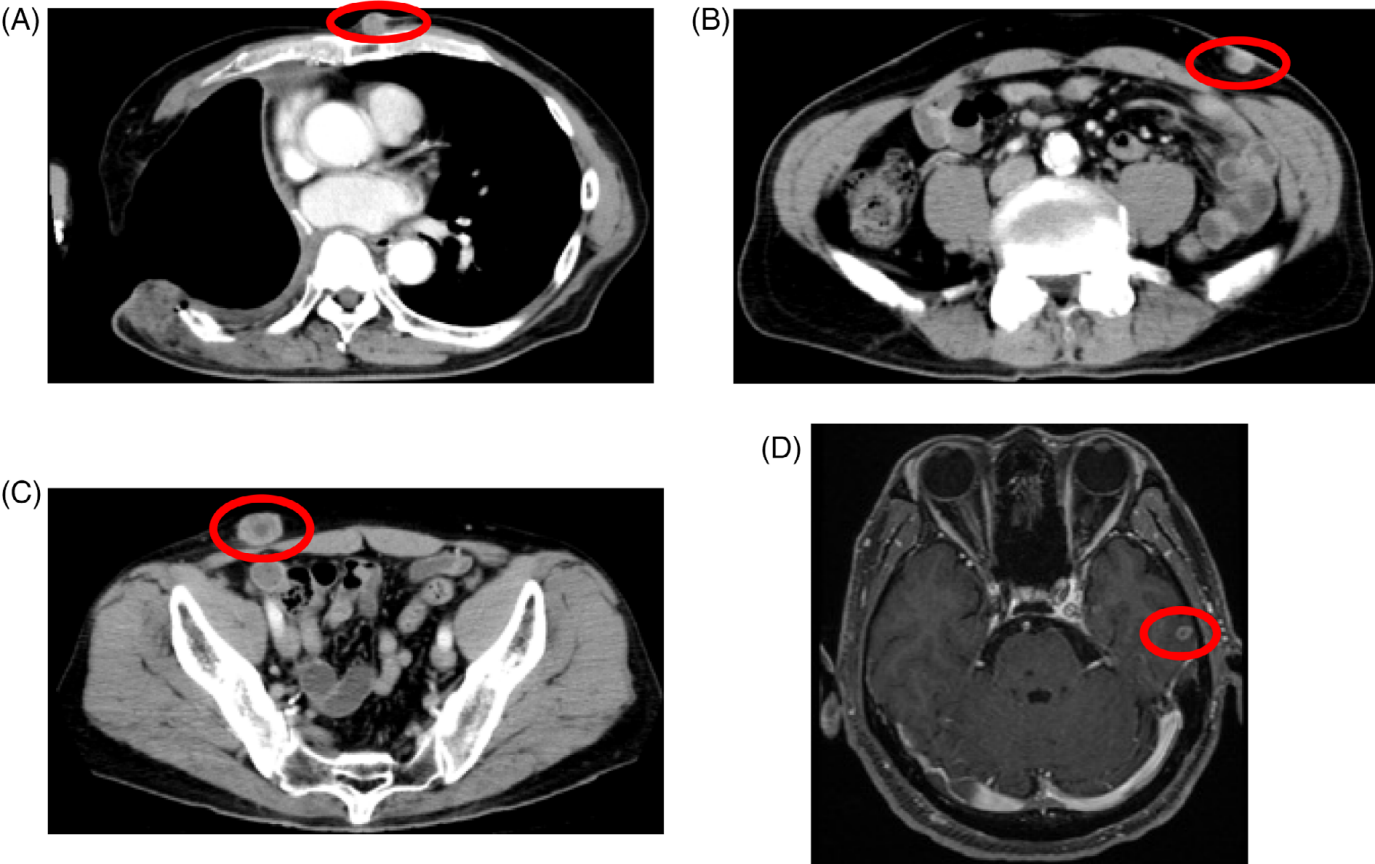
(A–C) Contrast‐enhanced CT and (D) MRI at the time of postoperative recurrence. Thoracoplasty had been performed due to postoperative empyema. A subcutaneous mass in the precordium and multiple subcutaneous metastases were observed on chest and abdominal CT. MRI showed brain metastasis

A 65‐year‐old male presented to our hospital with a cough for 2 months. Chest X‐ray revealed a tumor shadow in the right middle lung field. He had smoked two packets of cigarettes a day for 47 years and was diagnosed with prostatic cancer at 56 years. The Eastern Cooperative Oncology Group Performance Status was 1. Blood tests showed normal ranges for white blood cells (8020/μL), red blood cells (540 × 10^4^/μL), platelets (23.9 × 10^4^/μL), carcinoembryonic antigen (1.5 ng/mL), cytokeratin 19 fragments (5.7 ng/mL), squamous cell carcinoma‐associated antigen (0.9 ng/mL), pro‐gastrin‐releasing peptide (36.3 pg/mL), and prostate‐specific antigen (1.88 ng/mL). A tumor was observed in the right upper lobe on chest X‐ray (Figure 1(A)) and contrast‐enhanced computed tomography (CT) (Figure 1(B), (C)). No other distant metastases were detected and, thus, the patient underwent right pneumonectomy. The diagnosis was right upper lobe LCNEC pT3N1M0 stage IIIA. He underwent thoracoplasty due to postoperative empyema. Four months after right pneumonectomy, a subcutaneous mass in the precordium and multiple subcutaneous metastases were observed on chest and abdominal CT (Figure 2(A)–(C)). Furthermore, magnetic resonance imaging (MRI) showed brain metastasis (Figure 2(D)).

**FIGURE 3:**
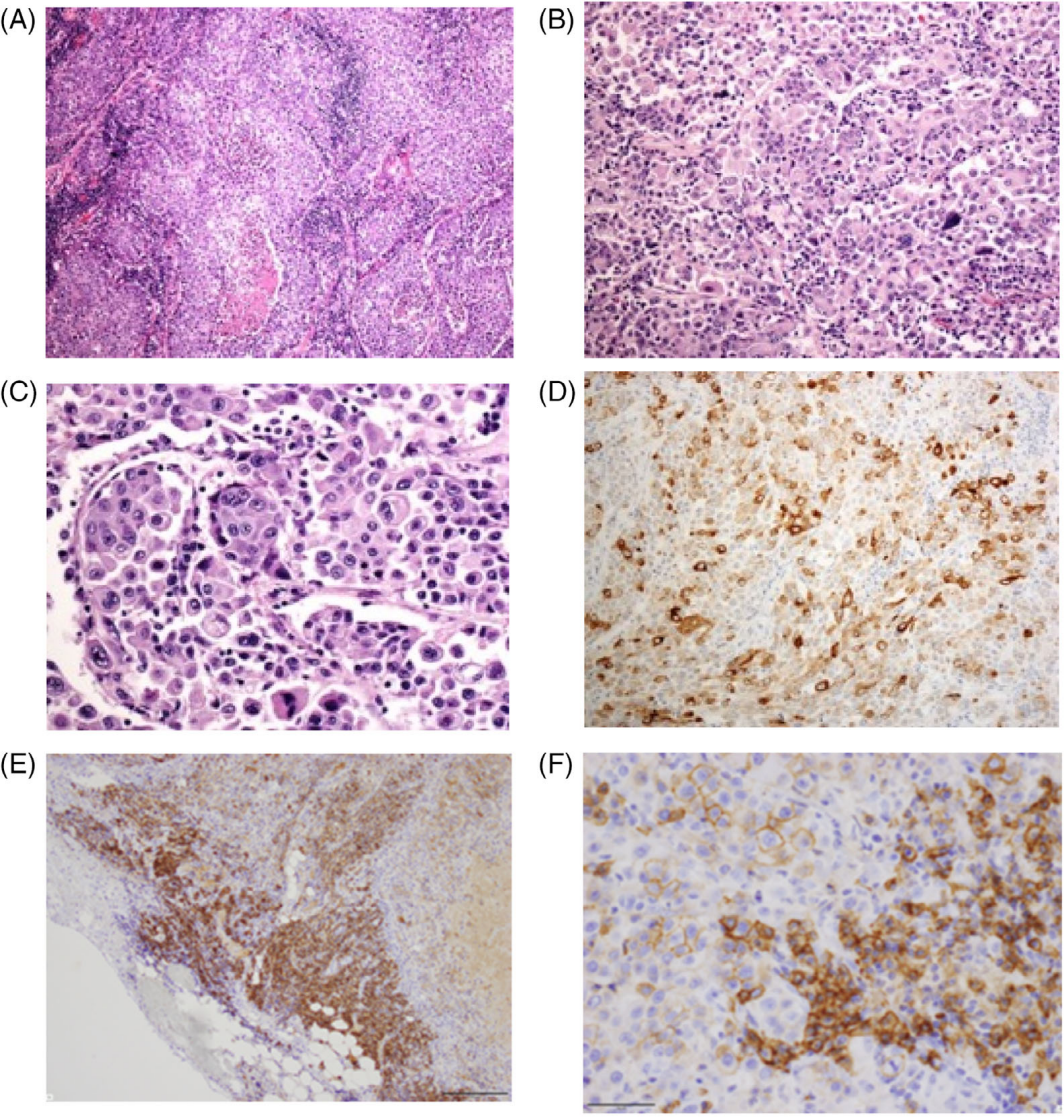
(A–C) Hematoxylin and eosin staining showed that tumor cells included atypical large cells and prominent nucleoli. Tumor cells also exhibited neuroendocrine architectural features, such as palisading features and necrotic areas (A, magnification ×40; B, magnification ×200; C, magnification ×400). (D–F) Immunostaining showed that tumor cells were diffusely positive for (D) chromogranin (magnification ×200), (E) CD138 (magnification ×100), and (F) CD138 (magnification ×400)

Histopathological imaging revealed that tumor cells had sheets and nests of atypical large cells with prominent nucleoli. Tumor cells exhibited neuroendocrine architectural features (Figure 3(A)–(C)). Immunostaining was negative for CD56 and synaptophysin, but positive for chromogranin, and the final diagnosis was LCNEC (Figure 3(D)). The primary cells infiltrating the subcutaneous tumor were CD3‐positive T cells and CD138‐positive plasma cells (Figure 3(E), (F)).

**FIGURE 4:**
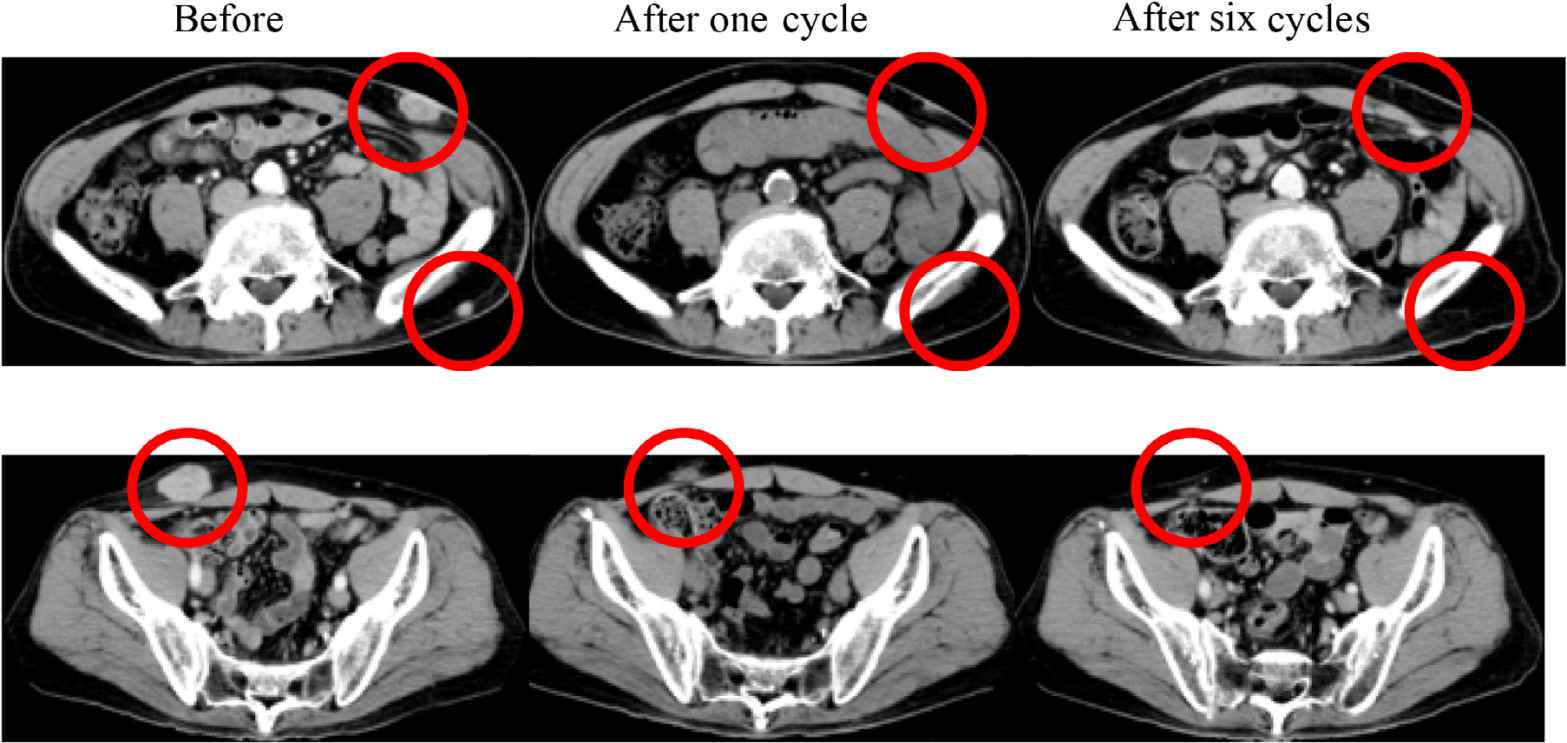
CT after pembrolizumab monotherapy. The tumor had completely disappeared after six cycles

Chemotherapy with carboplatin plus etoposide was initiated. Subcutaneous tumors had increased and new brain metastatic lesions had developed after two cycles. A genetic analysis of the original tumors revealed that epidermal growth factor receptor and anaplastic lymphoma kinase were negative, and the PD‐L1 expression rate in tumor cells was 40% (22C3 clones). Pembrolizumab was initiated as a second‐line treatment. The shrinkage of subcutaneous masses was noted after one cycle, and the tumor had completely disappeared after six cycles (Figure 4). Treatment was continued for approximately 2 years. This response has been maintained for 4 years and is still ongoing.

## 3 DISCUSSION

We encountered a rare case showing the CR of pulmonary LCNEC to pembrolizumab monotherapy. This response has been maintained for 4 years and is still ongoing. To the best of our knowledge, the CR of pulmonary LCNEC to immune checkpoint inhibitors (ICI) is extremely rare, with only three case reports in the literature.^5^, ^6^, ^7^

LCNEC is classified as pulmonary neuroendocrine carcinoma by the 2015 World Health Organization classification. The prognosis of pulmonary LCNEC is poor and standard therapy has not yet been established. However, ICI were effective in several cases of pulmonary LCNEC.^4^, ^8^, ^9^, ^10^ Our case and previous case reports are summarized in Table 1. The mean age of patients was 55 years, and only two cases were women. Many cases were heavy smokers and PD‐L1 expression levels were not high. Two cases were treated with pembrolizumab, six with nivolumab, and one with nivolumab plus ipilimumab. The effects of ICI were CR in four patients, a partial response (PR) in three, and stable disease (SD) in two. In a retrospective cohort of 11 patients with LCNEC treated with ICI, the response rate was 9.1% (one patient) and median progression‐free survival was 2.7 months.^11^

**TABLE 1:** LCNEC patients who responded to immune checkpoint inhibitors

No	Author [Ref.]	Age (years)	Sex	Stage	Smoking history (peck‐years)	PD‐L1: TPS	Chemotherapy	ICIs	Response
1	Wang [4]	64	M	Recurrence	40	<1%	3rd line	Pembrolizumab	PR
2	Mauclet [5]	41	F	IIIB	Current smoker	1–5%	2nd line	Nivolumab	pCR
3	Chauhan [6]	57	Unknown	IV	Unknown	Unknown	3rd line	Nivolumab	CR
4	Chauhan [6]	39	F	IV	Unknown	Possitive	2nd line	Nivolumab	SD
5	Zhang [7]	54	M	Recurrence	40	<1%	1st line	Nivolumab	CR
6	Takimoto [8]	62	M	IV	80	<1%	3rd line	Nivolumab	SD
7	Oda [9]	60	M	Recurrence	100	Unknown	3rd line	Nivolumab	PR
8	Qin [10]	49	M	IV	Unknown	<1%	2nd line	Nivolumab and ipilimumab	PR
9	Our case	65	M	Recurrence	94	40%	2nd line	Pembrolizumab	CR

Abbreviations: CR, complete response; PD‐L1, programmed death ligand 1; PR, partial response; pCR, pathological complete response; SD, stable disease; TPS, tumor proportion score.

PD‐L1 was previously reported to be expressed in approximately 10%–20% of pulmonary LCNEC cases.^12^, ^13^ However, no correlation has been reported between PD‐L1 expression levels and OS with ICI. On the other hand, although it was not measured in the present study, pulmonary LCNEC harbors a high mutation burden.^13^ Immune cell infiltration is more frequent in pulmonary LCNEC versus SCLC (58% vs. 23%), as is the expression of PD‐L1 on immune cells (46% vs. 23%).^13^ In the present case, the primary cells infiltrating the tumor were CD138‐positive plasma cells. A similar case report was published for lung cancer.^14^ Tumor‐infiltrating plasma cells are regarded as a favorable prognostic factor in lung cancer.^15^, ^16^ However, this is merely suggestive. In various cancer types, the presence of tertiary lymphoid structures has been associated with longer disease‐free and overall survival in patients treated with ICI.^17^ In contrast, the suppression of antitumor effects by plasma cell subgroups has been demonstrated.^18^ Further studies are needed to identify predictive biomarkers of ICI for LCNEC.

Immunotherapy is expected to improve the prognosis of patients with pulmonary LCNEC.^19^ According to the findings of several phase II studies, ICI are a promising therapeutic option for pulmonary LCNEC.^20^ Therefore, ICI may be used to treat patients with pulmonary LCNEC.

## 4 CONCLUSION

We encountered a rare case showing the CR of pulmonary LCNEC to pembrolizumab monotherapy. ICI are a promising treatment option for pulmonary LCNEC.

## CONFLICT OF INTEREST

The authors have stated explicitly that there are no conflicts of interest in connection with this article.

## AUTHOR CONTRIBUTIONS

All authors had full access to the data in the study and take responsibility for the integrity of the data and the accuracy of the data analysis. Conceptualization, N.H., E.T.; Methodology, M.K., Y.O.; Investigation, Y.K.; Formal Analysis, H.M.; Resources, N.H., H.H.; Writing&Original Draft, N.K.; Writing&Review Editing, E.T.; Visualization, K.N.; Supervision, T.S., S.S., F.O.

## ETHICS STATEMENT

Informed consent was obtained to publish this report.

## Data Availability

The data that support the results of this study are available from the corresponding author upon reasonable request.
